# Construction of a *Catsper1* DNA Vaccine and Its Antifertility Effect on Male Mice

**DOI:** 10.1371/journal.pone.0127508

**Published:** 2015-05-18

**Authors:** Qiong Yu, Xiao-Qin Mei, Xiao-Fang Ding, Ting-Ting Dong, Wei-Wei Dong, Hong-Gang Li

**Affiliations:** 1 Family Planning Research Institute/Center of Reproductive Medicine, Tongji Medical College, Huazhong University of Science and Technology, Wuhan, China; 2 Centre of Reproductive Medicine, Union Hospital, Tongji Medical College, Huazhong University of Science and Technology, Wuhan, China; 3 Wuhan Tongji Reproductive Medicine Hospital, Wuhan, China; Federal University of Pelotas, BRAZIL

## Abstract

Cation channel of sperm 1 (CATSPER1) is a unique sperm cation channel protein, and essential for sperm function and male fertility. CATSPER1 exclusively expresses in meiotic and postmeiotic spermatogenic cells, thus belongs to the spermatogenesis-specific antigen that escape central tolerance. We have previously demonstrated the immunocontraceptive potential of its transmembrane domains and pore region, and reported the antifertility effects of its B-cell epitopes on male mice. Aiming to develop DNA vaccine targeting CATSPER1 for male contraception, here the whole open reading frame of mouse *Catsper1* was cloned into the plasmid pEGFP-N1 to obtain a DNA vaccine pEGFP-N1-Catsper1. The vaccine was confirmed to be transcribed and translated in mouse N2a cell *in vitro* and mouse muscle tissue *in vivo*. Intramuscular injection with the vaccine on male mice induced specific immune reaction and caused significant inhibition on sperm hyperactivated motility and progressive motility (*P*<0.001 for both), and consequently reduced male fertility. The fertility rate of experimental group was 40.9%, which was significant lower (*P*=0.012) than control group (81.8%). No significant change in mating behavior, sperm production and histology of testis/epididymis was observed. Given that *Catsper1* exhibits a high degree of homology among different species, *Catsper1* DNA vaccine might be a good strategy for developing an immunocontraceptive vaccine for human and animal use.

## Introduction

The world population is still growing, even though the present contraceptives have made some contributions to controlling the growth rate. It is predicted that the total world population will reach 8 billion by 2020 and 8.9 billion by 2050 [1]. At present, the effective contraceptive methods for men are limited to condoms and vasectomy [2], which apparently cannot meet the different preferences of people. Moreover, most men express their willingness to carry contraceptive responsibility if there were more available contraceptives for men [3]. In this sense, it is a world-wide mission to develop new male contraceptives.

Researches on the relationship between immune infertility and antisperm antibody enlighten immunocontraception targets on sperm-specific proteins [4, 5]. The mechanism is that enough specific antibodies will be induced by sperm antigen and block the function of corresponding sperm proteins. Therefore, sperm proteins for immunocontraception should be located on the sperm surface and are accessible to the antibodies induced by the vaccines [6]. Immunocontraception has good application foreground and is considered to be the best way of fertility inhibition. It is relatively long lasting, reversible and easy to use; most importantly, it does not affect libido [2, 7, 8]. Many immunocontraceptive vaccines target different sperm membrane proteins have been experimented on animals, and some showed a good antifertility potential [7, 9]. Besides the contraceptive potential for human use, the immunocontraception strategy may also become an alternative and more humane means to control overpopulation of feral or domestic animals [10, 11].

One potential candidate of sperm membrane protein for immunocontraceptive is cation channel of sperm 1 (CATSPER1). It is a member of the unique sperm cation channel protein family, which is exclusively expressed in spermatogenic cells at meiosis and post-meiosis stage [12, 13]. CATSPER1 is primarily located to principal piece of the sperm tail, and is required for sperm hyperactivation and male fertility [12, 14, 15]. The mutant male mice with genotype of *Catsper1* ^-/-^ are normal in body weights, testis weights and sperm counts, but are completely infertile as the result of impairments in sperm motility and ability to penetrate the egg outer layers [12]. There are also some reports on human male infertility caused by mutations in *CATSPER1* [16]. Based on its restricted expression patterns and indispensable role in fertilization, CATSPER1 is predicted to meet the criteria for developing human immunocontraceptive vaccines [17], possessing a good contraceptive potential and low side effects. As a preliminary exploration, we have founded that the antibody against transmenbrane domains and pore region of CATSPER1 can significantly inhibit sperm progressive motility, hyperactivated motility and fertilization *in vitro* [18]. Then we tested the antifertility effects of two B-cell epitopes in its transmembrane domains, and significant antifertility effects were observed on male mice [19]. These results prompted us to develop a *Catsper1* DNA vaccine for male contraception.

DNA vaccines have been used in various fields. In the field of male contraception, DNA vaccines targeting sperm membrane proteins could potentially become an ideal immunocontraceptive [20]. Some plasmid DNA vaccines have shown potential antifertility effect on male or female animals [21–26]. Compared with protein vaccines, DNA vaccines possess many obvious advantages. It is more heat stable, easy to produce and inexpensive [26]. The conformation and antigenicity of the antigenic protein encoded by DNA vaccines are identical to native antigen, and it has no potential pathogenicity [22]. In addition, it induces a long-lived humoral response, and the antibody has a greater avidity than protein-raised antibody [27–30]. DNA vaccines can also induce cellular responses [22, 31–33].

CATSPER1 belongs to spermatogenesis-specific antigens that are completely absent from the antigenic repertoire until the time of puberty and escape central tolerance. We proposed that the exogenously expressed CATSPER1 could induce specific antibody, which is physiologically avoided due to the immune-privileged status of the testis [34]. Herein we constructed a *Catsper1* DNA vaccine by inserting the whole open reading frame (ORF) of mouse *Catsper1* into eukaryotic expression plasmid pEGFP-N1, and evaluated its antifertility effect on male mice, aiming to develop a DNA vaccine targeting CATSPER1 for male contraception and management of animal populations.

## Materials and Methods

### Amplification of the ORF of *Catsper1*

**Fig 1:**
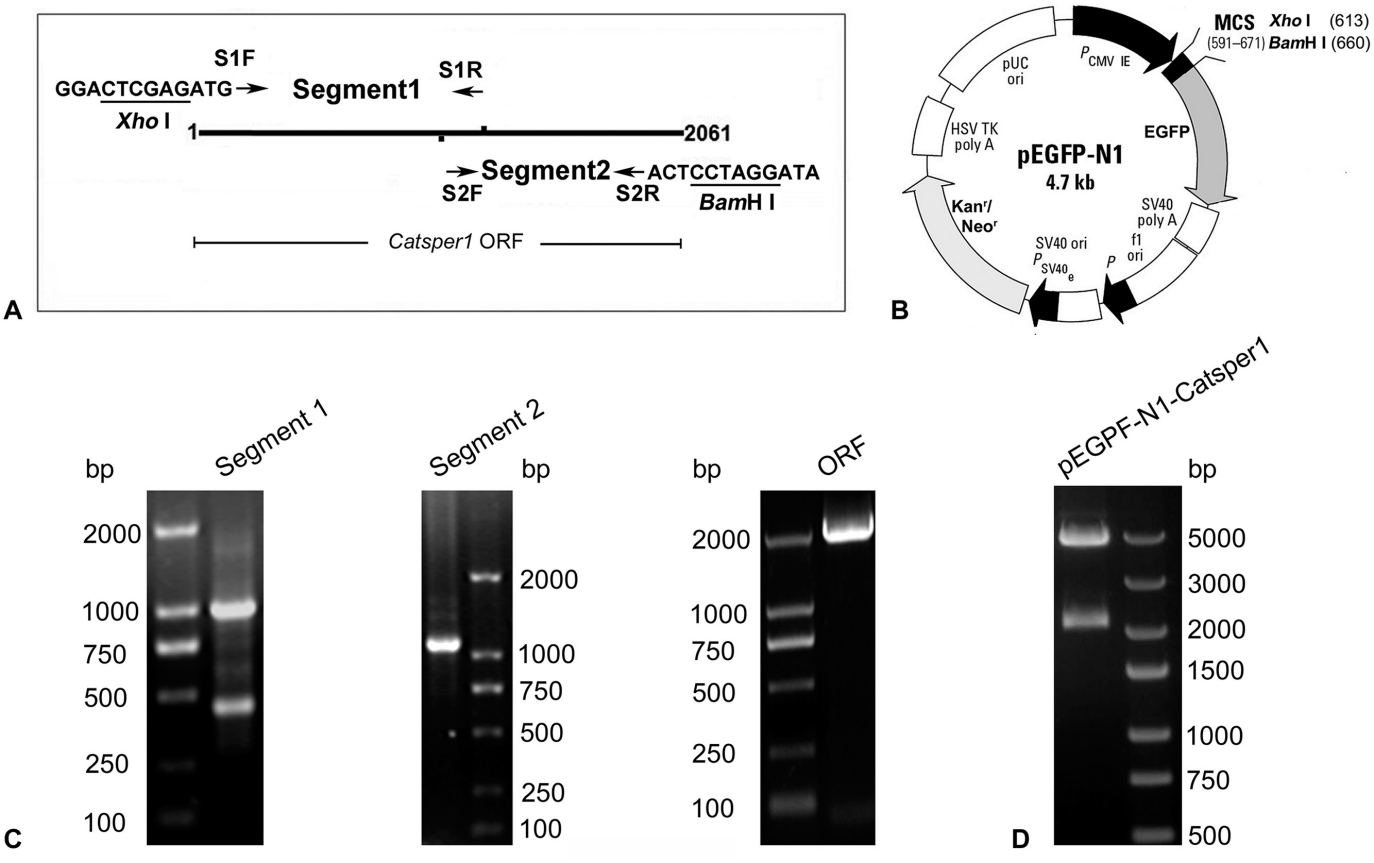
Construction and identification of the DNA vaccine plasmid pEGFP-N1-Catsper1. (A) The amplification of the whole *Catsper1* ORF by recombinant PCR. The sequences marked with underline are the restriction enzyme sites of *Xho* I and *Bam*H I. (B) Schematic diagram of pEGFP-N1 plasmid. EGFP was used as a report gene, and Kanr/Neor was a kanamycin resistance gene and used for colony screening of *E*.*coli* that transformed with plasmid. (C) Agarose gel electrophoresis of the recombinant PCR products. PCR products are, in order, the former half (Segment 1) of *Catsper1* ORF with restriction enzyme site (1107 bp), the latter half (Segment 2) of *Catsper1* ORF with restriction enzyme site (1106 bp) and the whole *Catsper1* ORF with restriction enzyme sites (2079 bp). (D) Agarose gel electrophoresis of restriction enzyme digestion products of recombinant plasmid pEGFP-N1-Catsper1.

The size of mouse *Catsper1* ORF is 2061 bp and we failed to amplify it with single traditional reverse transcription polymerase chain reaction (RT-PCR) from mouse testicular cDNA. Then, we applied a two-step recombinant PCR strategy (Fig 1A). In the first step, the former half (Segment 1) and the latter half (Segment 2) of *Catsper1* ORF were amplified from mouse testicular cDNA with primer set S1F, S1R and primer set S2F, S2R respectively. The two PCR products were then used as templates for the second step, through which the whole ORF was amplified by recombination PCR with primers S1F and S2R. The primer sequences were as follows: 5’-GGACTCGAGATGGATCAATCTTCAAGGAGG-3’ (S1F), 5’-GACTGTGTTGAGGCAGACCACGAT-3’(S1R), 5’-CCGGGAATATCTTCCAATTGCTATG-3’ (S2F), 5’-ATAGGATCCTCACTTCCCGTAGTCGTCGTC-3’ (S2R). Annealing temperatures for PCR were 66°C for S1F and S1R, 53°C for S2F and S2R, and 66°C for S1F and S2R.

### Construction and preparation of recombinant plasmid pEGFP-N1-Catsper1

The ORF of mouse *Catsper1* was inserted into the pEGFP-N1 plasmid (Clontech, CA, USA; Fig 1B) to obtain a recombinant plasmid pEGFP-N1-Catsper1, by digestion with *Xho* I and *Bam*H I and ligation with T4 DNA ligase according to the manufacturers’ instructions. *E*.*coli* strain DH5α was transformed with the plasmid pEGFP-N1-Catsper1 and cultured overnight in the LB liquid medium which contains kanamycin. The recombinant plasmid was extracted from *E*.*coli* and confirmed by restriction enzymes digestion and DNA sequencing. Large amount of plasmid were prepared for later experiments using EndoFree Plasmid Maxi Kit (Biomiga, CA, USA) according to the manufacturer’s instructions. Plasmid concentration was adjusted to 0.5g/L with phosphate-buffered saline (PBS) for the injection use on mice.

### Expression of pEGFP-N1-Catsper1 in mouse N2a cells *in vitro*

Recombinant plasmid pEGFP-N1-Catsper1 was transfected into mouse N2a cells cultured in six-well plates by Lipofectamine 2000. At 24 hours after the transfection, expression of *Catsper1* mRNA was detected with RT-PCR. Total RNA was extracted from the cultured cells using TRIzol according to the manufacturer’s instructions. To avoid non-specific amplification, RNA was subjected to DNase I treatment to remove any contaminating DNA, then cDNA was synthesized with the RevertAid First Strand cDNA Synthesis Kit according to the manufacturers’ instructions. Oligo(dT) primers were used during the cDNA synthesis. The primers used for *Catsper1* amplification were as follows: 5’-TTTACCTGCCTCTTCCTCTTCT-3’ (Forward), 5’-ACCAGGTTGAGGAAGATGAAGT-3’ (Reverse). The annealing temperature is 58°C and the amplification size is 227 bp. At 48 hours after the transfection, the expression of CATSPER1 protein was identified by the observation of enhanced green fluorescent protein (EGFP) fluorescence under a laser confocal microscope. Since the recombinant plasmid can express fusion protein EGFP-CATSPER1 once it is transfected to cells or injected to animals.

### Expression of pEGFP-N1-Catsper1 in mouse muscle *in vivo*

The male and female BALB/c mice (aged 8–9 weeks) were obtained from the Center for Disease Control and Prevention of Hubei Province in China (Grade SPF, Certificate No. scxk 2008–0005), and were housed in a temperature- and humidity-controlled environment with 12-hour light/12-hour dark cycle. All experiments with the animals in this study were performed according to the National Institutes of Health Guiding Principles in the Care and Use of Animals, and the protocols were approved by the Reproductive Medicine Review Board of Tongji Medical College.

To test whether the recombinant plasmid pEGFP-N1-Catsper1 could express *in vivo* and induce specific immune reactions, male mice were injected with pEGFP-N1-Catsper1 (3 mg/kg of body weight) at the thigh muscles of one hind limb. The mice subsequently were given two booster injections by the same method at 2-week intervals. Total RNA was extracted from the tissue at injection site 12, 24 and 48 hours after the first injection respectively, and subjected to the detection of *Catsper1* mRNA expression. RNA isolation, DNase I treatment, and RT-PCR were performed as mentioned above. The tissue at injection site was collected for frozen section 24, 48 and 72 hours after the first injection respectively, and EGFP was examined under a laser confocal fluorescence microscope. Blood was collected to perform the following enzyme-linked immunosorbent assay (ELISA).

### ELISA

ELISA was used to detect the induced antibodies before and 1, 3, 5 weeks successively after the first injection. Blood was stored at 4°C for 30 min, and sera samples were isolated by centrifugation and stored at -70°C. The titers of IgG antibody against CATSPER1 protein in the serum were determined as previously described [19]. Two B-cell epitopes in transmembrane domains of CATSPER1 (P34 and Pp6), which were previously predicted using BCEPRED program were used as coating antigen. The first one is a 18-mer peptide between transmembrane domain 3 and 4 (P34), and the other one is a 15-mer peptide between the pore region and transmembrane domain (Pp6), both were identified to possess good antifertility potential in our previous experiments [19]. All sera samples were diluted to 1:100, and horseradish peroxidase-labeled goat anti-mouse antibody was diluted to 1:5000.

### Immunofluorescence

Immunofluorescence was performed to determine the specificity of antiserum. Normal sperm suspension from caudal epididymis was dropped onto slides, air-dried and then fixed with 4% paraformaldehyde for 30 minutes at roomtemperature. The slides were washed three times with PBS and then blocked with normal goat serum for 30 minutes. After three washings with PBS, sperm were incubated with antiserum from immunized mice over night at 4°C, and serum from unimmunized mice were used as negative control. The slides were then washed three times and incubated with secondary antibody (1:500 diluted fluorescein isothiocyanate conjugate-labeled goat anti-mouse antibody) for 1 hour at 37°C. The slides were washed three times and examined under a laser confocal fluorescence microscope.

### Mating trial of immunized mice

After the confirmation of the correct construction and expression of the plasmid pEGFP-N1-Catsper1, as well as its antibody induction potential in mice, its antifertility effect on male mice was then evaluated. Male mice were randomly divided into two groups with 11 mice in each group. One group was immunized with pEGFP-N1-Catsper1 and the other group (control group) with pEGFP-N1 plasmid, both administered the same dosage as mentioned above. The mice subsequently were given two booster injections refering to the same method at 2-week intervals. Mating trial was performed 1 week after final immunization by a female to male ratio was 2:1. Mated females were examined within 8 hours from cohabitation for the vaginal plug, which is considered as the mark of successful mating. Mated females were euthanized to examine the embryos in their uteri 14 days later.

### Histology analysis and sperm function assessment

Males were euthanized after mating trial, one side of the testis and epididymis were paraffin embedded, then stained with hematoxylin and eosin. The other side was used for the assessments of sperm density, motility and hyperactivated motility. A 100 μL droplet of IVF-30 (Vitrolife, Gothenburg, Sweden) covered with mineral oil in a petridish was equilibrated in incubator at 37°C, 5% CO_2_ over night before use. Caudal epididymis was placed under the mineral oil, and sperm were extracted and dispersed in IVF-30 according to the methods described by Chang H et al [35]. After 10 minutes, the swim-up suspension was used for the analysis of sperm density and motility as previously described [19]. Spermatozoa with progressive motility (forward movement), non-progressive motility (swing in the same spot) and no movement were calculated. The sperm suspension was diluted to the proper concentration of (5–10)×10^6^ sperm/mL with IVF-30 and further incubated for 1.5 hours after collection, and then used for the analysis of hyperactivation, which is characterized by high-amplitude, asymmetrical flagellar beating and circular or helical swimming trajectories [36]. IVF-30 was bicarbonate buffered medium containing human serum albumin and calcium, in which sperm hyperactivation could be initiated without adding other inducers. Spermatozoa with hyperactivated and non-hyperactivated motility were determined subjectively under the light microscope and at least 5 visions were calculated.

### Statistical analysis

Comparisons of mating rate and fertility rate were performed using chi-squared test. Mann-Whitney U test was used to compare the mean litter size, sperm density, motility and hyperactivated motility. All statistical tests were two-tailed and a *P* value <0.05 was considered statistically significant.

## Results

### Construction of the plasmid pEGFP-N1-Catsper1

The ORF of mouse *Catsper1* was amplified by a two-step recombinant PCR. In the first step, two bands approximately 1.1 kb were detected in the RT-PCR products, which were consistent with the fragment size of the former half of the *Catsper1* ORF plus restriction enzyme site (Segment 1, 1107 bp) and latter half of the *Catsper1* ORF plus restriction enzyme site (Segment 2, 1106 bp). In the second step, a band approximately 2.1 kb was detected in the PCR product, which was consistent with the fragment size of the whole *Catsper1* ORF plus restriction enzyme sites (Fig 1C).

After the whole ORF of mouse *Catsper1* was integrated into pEGFP-N1 plasmid, the recombinant plasmid was transformed to *E*.*coli*. The extracted plasmid was successfully confirmed by restriction enzymes digestion and DNA sequencing. After digestion with *Xho* I and *Bam*H I, two bands approximately 2 kb and 5 kb were detected, which was consistent with the predicted size of the product (Fig 1D).

Large amount of the recombinant plasmid pEGFP-N1-Catsper1 was successfully recovered, with the OD_260_/OD_280_ ratio between 1.8 and 2.0. Its concentration was adjusted to 0.5 g/L for the subsequent injection use on mice.

### Expression of pEGFP-N1-Catsper1 in mouse N2a cells *in vitro*

**Fig 2:**
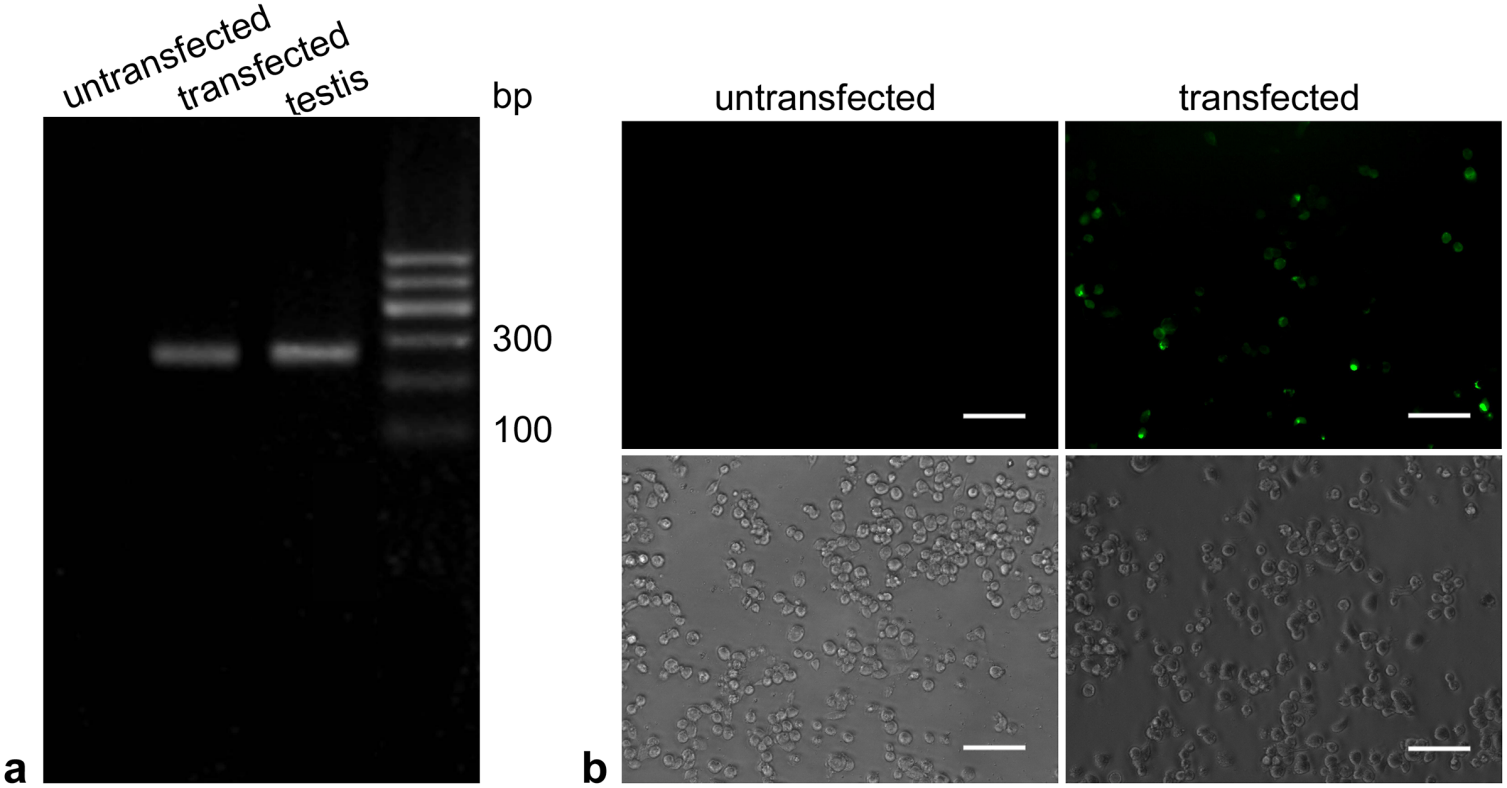
*In vitro* expression of pEGFP-N1-Catsper1 in mouse N2a cells. (A) mRNA expression detected by RT-PCR. Templates were cDNA from untreated mouse N2a cells, N2a cells transfected with pEGFP-N1-Catsper1 and mouse testis (positive control) respectively. (B) Expression of EGFP-CATSPER1 fusion protein in N2a cells transfected with pEGFP-N1-Catsper1. Scale bar = 100 μm.

The recombinant plasmid pEGFP-N1-Catsper1 was transfected into mouse N2a cells to test whether could be expressed in eukaryotic cells. At 24 hours after transfection, RT-PCR results showed that a specific band was detected in the pEGFP-N1-Catsper1 transfected cells, demonstrating the expression of *Catsper1* mRNA (Fig 2A). At 48 hours after transfection, strong green fluorescence was observed on the pEGFP-N1-Catsper1 transfected cells, demonstrating the expression of the fusion protein EGFP-CATSPER1. No fluorescence was observed on untreated cells (Fig 2B).

### Expression of pEGFP-N1-Catsper1 in mice

**Fig 3:**
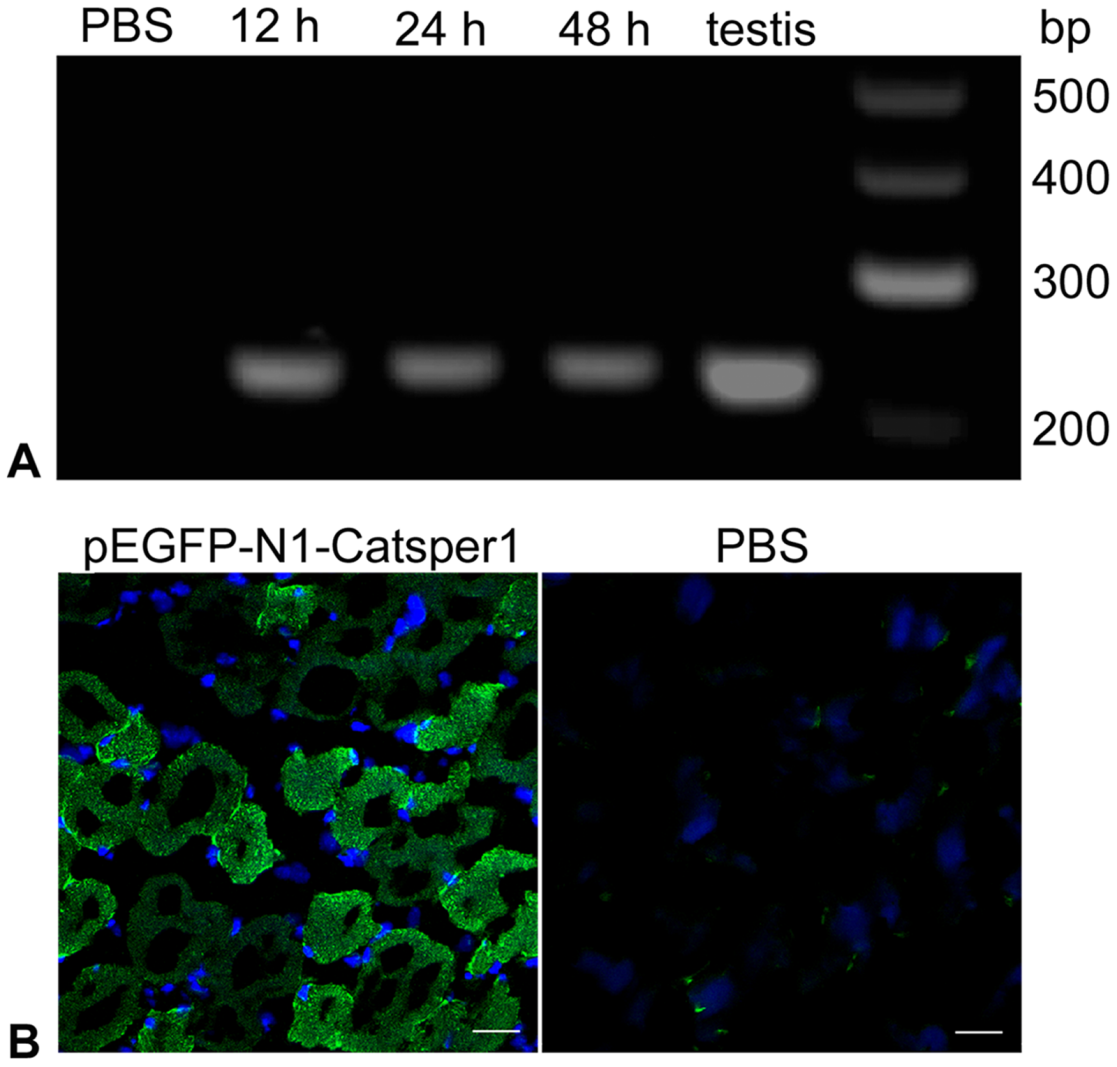
*In vivo* expression of pEGFP-N1-Catsper1 in mouse muscle tissue. (A) *Catsper1* mRNA expression detected by RT-PCR. Lane PBS: muscle tissue of PBS-injected mice; Lane 12 h to Lane 48h: muscle tissue of pEGFP-N1-Catsper1-injected mice 12, 24 and 48 hours after first injection respectively; Lane testis: mouse testis (positive control). (B) *In vivo* expression of EGFP-CATSPER1 fusion protein in the mouse muscle tissue. Scale bar = 20 μm.

The recombinant plasmid pEGFP-N1-Catsper1 was also injected into male mice at the thigh muscles of one hind limb to confirm it can be expressed *in vivo* and induce specific immune reactions. The *Catsper1* mRNA was detected in the muscle tissue injected with pEGFP-N1-Catsper1 at 12, 24 and 48 hours after first injection (Fig 3A). Strong green fluorescence, which demonstrated the expression of EGFP-CATSPER1 fusion protein, was observed on muscle cells in pEGFP-N1-Catsper1-injected mice at 24, 48 and 72 hours after the first injection (Fig 3B). Little spontaneous fluorescence was observed in PBS-injected mice.

### Antibody levels and specificity

**Fig 4:**
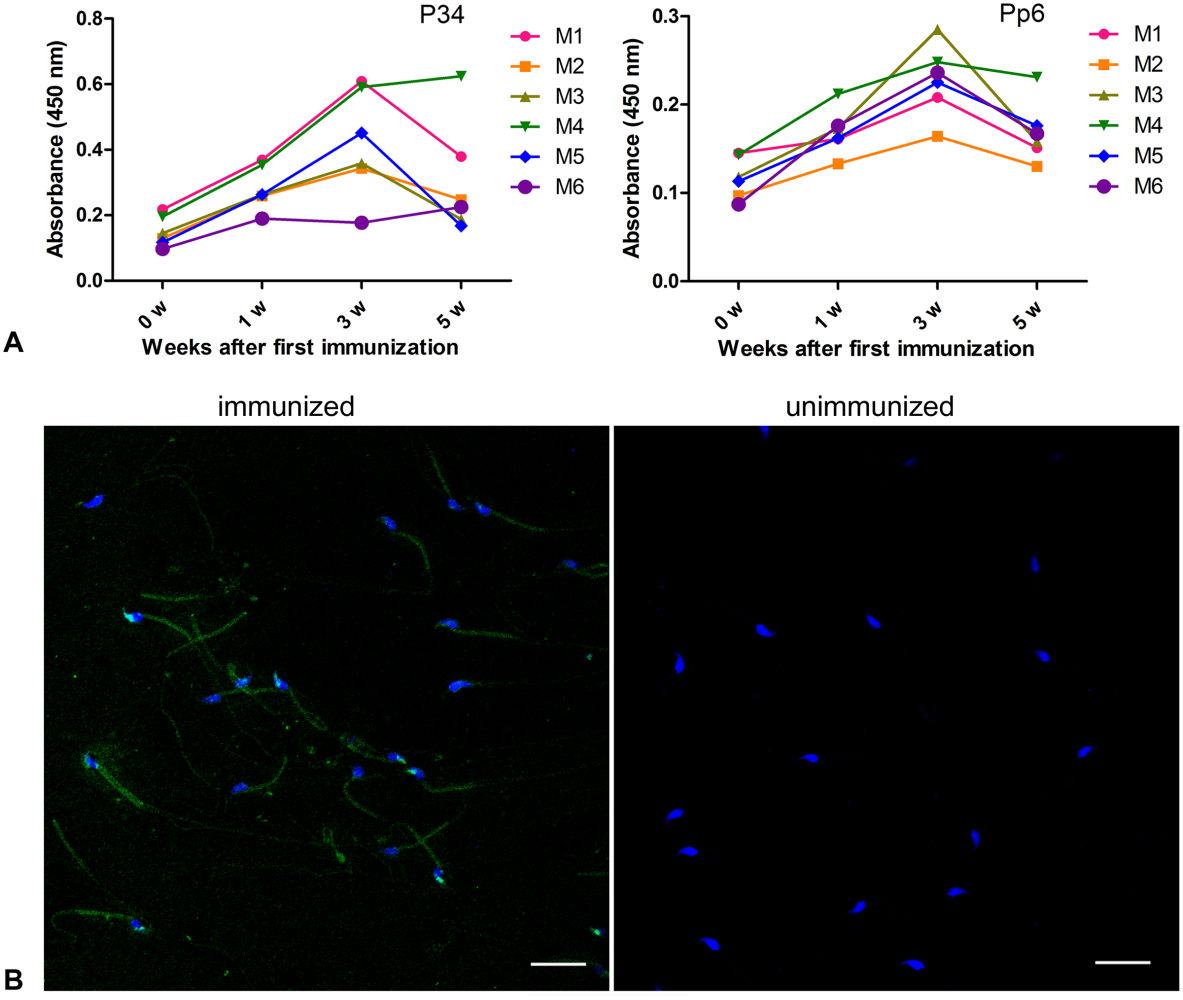
Antibody levels and specificity of pEGFP-N1-Catsper1-injected male mice. (A) Serum IgG antibody response of immunized male mice by ELISA. M1-M6 represent 6 individual mice. The 0 week on horizontal axis represents the time point before the first immunization, and others represent the weeks after first immunization. P34 and Pp6 were used as coating antigen in the ELISA assay respectively. (B) The specificity of antibodies in serum of immunized mice. Scale bar = 20 μm.

Blood was collected for ELISA to detect the induced antibodies before and 1, 3, 5 weeks after the first injection. As shown in Fig 4A, specific IgG antibodies against the transmembrane domains of CATSPER1 in serum samples of the pEGFP-N1-Catsper1-immunized male mice were detected. The antibody titers mainly increased with the time of immunization and reached the highest level at 3 weeks after the first immunization gradually, then showed a declining tendency. The absorbance at 450 nm was relatively higher using P34 as coating antigen.

To further evaluate the specificity of antibody in serum that responded to immunization, indirect immunofluorescence was performed. The results showed that antiserum from pEGFP-N1-Catsper1-immunized mice mainly bound to the sperm tail, which was same to the localization of CATSPER1 (Fig 4B). No immunofluorescence was observed in the negative control group.

### Antifertility effect

Immunized male mice were housed with female mice for 2 weeks after the final immunization, to test *in vivo* fertility. As shown in Table 1, an obvious reduction of male fertility was observed in pEGFP-N1-Catsper1-injected group, while the mating rates and mean litter size were similar with the pEGFP-N1-injected (control) group. The presence of vaginal plug was observed in 90.9% (20/22) and 95.5% (21/22) females mated with pEGFP-N1-Catsper1 and pEGFP-N1-injected mice, respectively, which showed no significant difference (*P* = 1.0). Among these plugged mice, 9 and 18 pregnant females were confirmed in the two groups respectively 14 days after coitum. Thus the fertility rate of pEGFP-N1-Catsper1-injected group was 40.9%, which was significant lower (*P* = 0.012) than the control group (81.8%).

**Table 1:** Fertility test of immunized male mice.

Group	Number	Matched female mice			
		Number	Mating rate^a^	Fertility rate^b^	Embryos^c^
pEGFP-N1-Catsper1	11	22	20/22	9/22^d^	10.9±0.9
pEGFP-N1	11	22	21/22	18/22	10.5±0.4

^a^ Shown are the number of mated mice/total number of mice.

^b^ Shown are the number of pregnant mice/total number of mice.

^c^ Represent in the form of means ± standard errors.

^d^ Statistically significantly different from the control group (*P*<0.05).

### Changes in sperm function and histology of testis and epididymis

**Fig 5:**
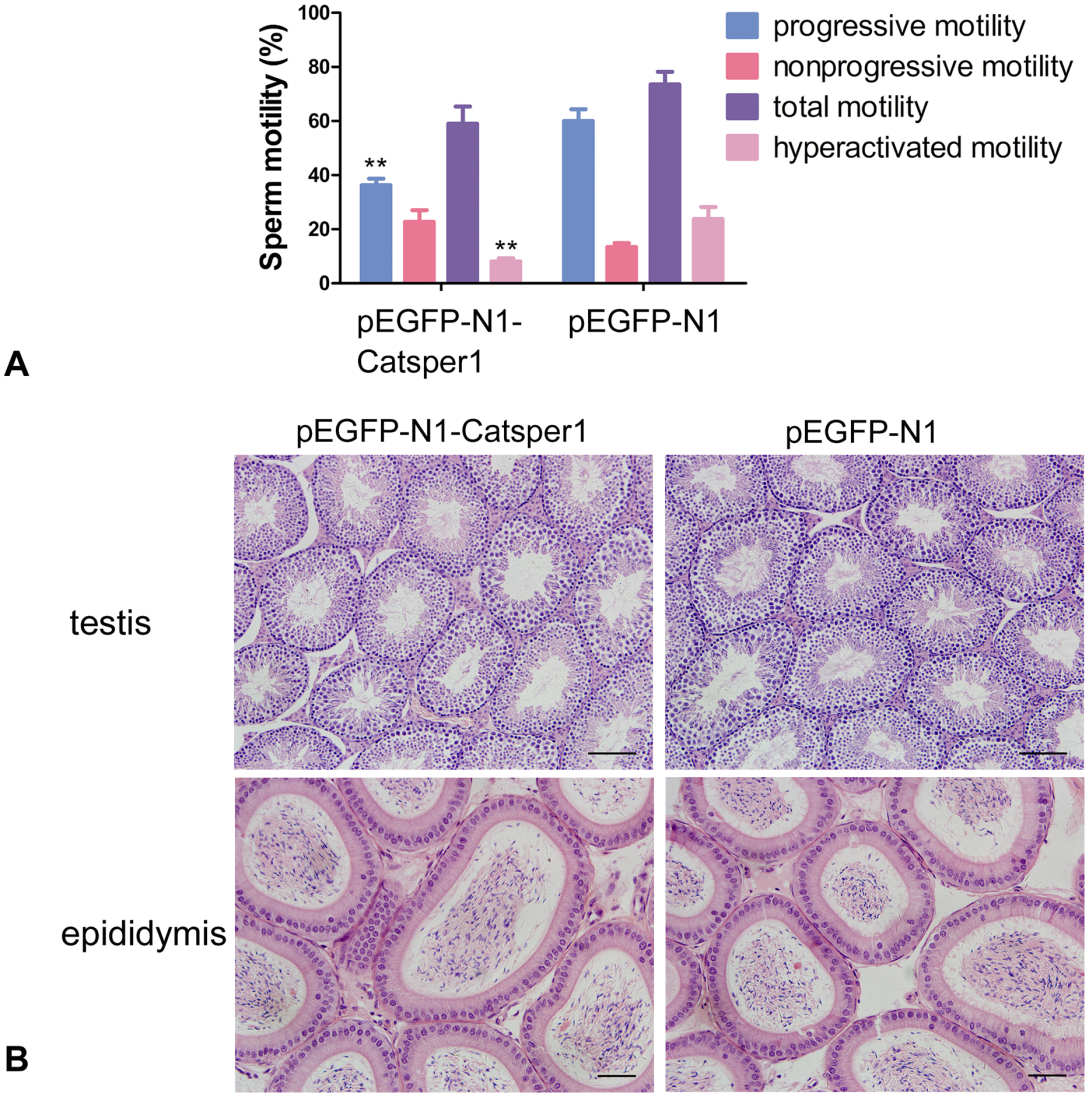
Changes in sperm motility and histology of testis and epididymis. (A) Sperm motility and hyperactivated motility of immunized mice. **P<0.001. (B) Histology of testis and epididymis of immunized mice. Scale bar of testis = 50 μm, scale bar of epididymis = 20 μm.

After mating trial, male mice were euthanized for epididymal sperm function assessment and testicular and epididymal histology analysis. Interestingly, our results showed that the sperm progressive motility and hyperactivated motility of pEGFP-N1-Catsper1-injected mice were inhibited, it were significantly lower (*P*<0.001 for both) than control mice, whereas the total motility showed no statistical difference between the two groups (Fig 5A). Additionally, the sperm concentration was (3.06±0.38) ×10^7^/mL in pEGFP-N1-Catsper1-injected mice, and (5.59±1.09) ×10^7^/mL in control mice, showing no significant difference. No pathologic change in histology of testis and epididymis was observed under the light microscope in pEGFP-N1-Catsper1-injected mice or the control group (Fig 5B).

## Discussion

CATSPER1 is a sperm-specific cation channel essential for male fertility [12]. Our previous results showed that immunization of BALB/c mice with B-cell epitopes of CATSPER1 inhibited male fertility significantly [19], which encouraged us to develop the *Catsper1* DNA vaccine. In the present study, the whole ORF of *Catsper1* was amplified from mouse testicular cDNA by recombinant PCR and successfully cloned to obtain plasmid pEGFP-N1-Catsper1. Before this plasmid was applied to the antifertility test on male mice, its correct transcription and translation in eukaryotic cells were confirmed *in vitro* and *in vivo*. *Catsper1* mRNA and strong green fluorescence of EGFP, which demonstrated the expression of EGFP-CATSPER1 fusion protein, was observed in the transfected N2a cells and injected muscle tissues. After intramuscular injection with the vaccine on male mice, specific immune reactions and significant reduction of male fertility were observed, showing a good contraceptive potential. To our knowledge, this represents the first complete research on the contraception potential of *Catsper1* DNA vaccine.

Gradually increased antibody against CATSPER1 was detected in male mice within 3 weeks after the first immunization with pEGFP-N1-Catsper1. The expression of CATSPER1 in mouse testis is initiated at meiosis stage [13], thus it belongs to spermatogenesis-specific antigens that escape central tolerance. Physiologically, immune reactions against CATSPER1 should be avoided mainly due to the immune-privileged status of the testis [34], whereas it could be induced by the exogenously expressed CATSPER1. Considering that in the live cell antibodies can only bind with the extracellular region, antibodies against two B-cell epitopes (P34 and Pp6) in the transmembrane domains of CATSPER1 that we previously identified [19], were analyzed. Antibodies against these two epitopes reached the highest level at 3 weeks after the first immunization and then showed a declining tendency, which was expected to remain stable for a time or decline slowly. The rapidly decreased antibody titers may atrribute to the short intervals between immunizations or some other factors. Time intervals are crucial for the vaccines’ efficacy, too short intervals may trigger weak immune responses, because booster antigens combine the antibodies triggered by the primary immunization and the antigen-antibody complexes will be cleared rapidly; moreover, too long intervals may also trigger weak immune response, memory B cells are long-lived, but not immortalized. In summary, properly increase the intervals between immunizations may be a good strategy to enhance the efficacy [37]. Although antibodies against the two epitopes showed similar changes during the immunization, the absorbance in ELISA was relatively higher using P34 as coating antigen, which was consistent with our previous ELISA experiments [19]. This difference between antibodies against P34 and Pp6 may be due to the different affinity between the antibody and antigen, or different immune potential of these two epitopes. Besides, there was also a difference in immunoreactive intensity among the different individuals, which might result from the individual variation in immunity and cause unwanted pregnancy in the individuals showed weaker immunoreactivity. To ensure the validity of immunocontraceptive vaccines, the evaluation of immune responses should be performed before scheduled immunization. To further evaluate the immune reactions, we also tried to detect the binding of antibodies to epididymal sperm in immunized mice by immunofluorescence as described previously [19]. However, no obvious fluorescence was observed (data not shown). Similar results were observed in our previous experiments and some other reports [19, 38]. It might result from the low amount of antibodies entered into reproductive tract and the poor sensitivity of the detection method. Comparisons of different techniques to detect antisperm antibodies also showed that low titer antibodies in some patients were undetectable by immunofluorescence assay but were detectable by other assays [39, 40]. Thus, we suppose that the amount of antibodies bound to sperm was not sufficient to be detected by immunofluorescence but was enough to inhibit the function of key proteins in sperm.

The mechanism of antifertility effects of pEGFP-N1-Catsper1 on male mice may consider that the specific antibody induced by the exogenously expressed CATSPER1 can inhibit the function of CATSPER1. Antibodies could reach CATSPER1 channel through the serum to the epididymis and reproductive fluids, given that antibodies can enter the male reproductive tract [38, 41–44]. CATSPER1 is required for male fertility due to its essential role in sperm hyperactivation [14, 15, 45, 46]. The hyperactivated motility of sperm from mice immunized with pEGFP-N1-Catsper1was significantly inhibited; consequently, the process of sperm-egg fusion should be interfered. In theory, the mating behavior and spermatogenesis will not be affected by antibodies against CATSPER1. Actually, no significant difference in mating rate, sperm concentration and histology of testis and epididymis was observed between the experimental group and control group in the present study and our previous work [19], suggesting that the *Catsper1* DNA vaccine can avoid some side effects associated with hormones. Furthermore, previous reported inconsistent results on the progressive motility of the *Catsper1* null sperm [12, 14]. In our present experiments, the progressive motility was significantly inhibited after the immunization with pEGFP-N1-Catsper1.

The antifertility effect of pEGFP-N1-Catsper1was relatively weaker compared with our previous results with B-cell epitopes of CATSPER1 [19]. We suppose it might be the result of insufficient immunoreactive intensity and binding of the antibodies to sperm. The fact that the immune effect of DNA vaccine is relatively poor on animals is a constrained bottleneck problem. Actually, researches on contraceptive vaccines targeting sperm-specific proteins are very limited. A handful of studies showed that the fertility rate of immunized male mice are 57.5%, 40.0% and 60% respectively without using adjuvant[25, 26, 47], which are similar to our results. More needs to be done to enhance the efficacy of DNA vaccines. In recent studies, more attention is paid on C3d, a complement C fragment. As a new molecular adjuvant, C3d can improve the immunogenicity of the antigen, reduce the activation threshold of B cells, and enhance the immunoreactive intensity [48, 49]. A previous research showed that immunization of mice with a recombinant model antigen, hen egg lysozyme (HEL), fused to two and three copies of murine C3d, resulted in 1000- and 10,000-fold enhancement in immune response, respectively [48]. Many reports have demonstrated that the immune efficacy of DNA vaccines contains antigen coding sequences were dramatically enhanced after fused with C3d molecular adjuvant [25, 47, 50]. Most importantly, it is safe and simple to use [25]. There are also some other methods, such as increase time intervals between immuniazations properly, take heterologous prime-boost regimen and so on [37].

Over all, the present study constructed a DNA vaccine, which can transcribe and translate the mouse *Catsper1* normally in male mice *in vivo* and induced specific immune reactions. The induced specific antibodies thus can block CATSPER1 channel and inhibit the fertility of male mice. Human *Catsper1* exhibits a high degree of homology with mouse. The transmembrane domains and pore region, which should be the targeting area for immunocontraception, even share 89% identity and 100% similarity [12]. The *Catsper1* DNA vaccine might be a strategy for the development of an immunocontraceptive vaccine for human and animal use. But it has a long way to go from basic research to clinical application of DNA vaccines for immunocontraception.
